# What leads to prejudice against homosexuality in China?—familism, filial piety, and gender role attitudes in a Confucian cultural context

**DOI:** 10.3389/fpubh.2026.1749610

**Published:** 2026-03-06

**Authors:** Hua Zhang, Yanyan Ouyang, Xiuxian Deng, Shulei Zhong, Chunyan Luo, Cheng Yang

**Affiliations:** 1 School of Politics and Public Administration, Guangxi Minzu University, Nanning, China; 2 School of Government, Shenzhen University, Shenzhen, China; 3 School of Public Policy and Management, Guangxi University, Nanning, China

**Keywords:** Confucian culture, familism, filial piety, gender role beliefs, homosexuality, sexual attitude

## Abstract

**Background:**

From a cultural perspective on research into homosexual identity, existing studies argue that the cultural foundations of Chinese attitudes toward homosexuality are deeply embedded in Confucian ethics, the family system, and a patriarchal gender order. Building on this foundation, this study adopts “family-based moral regulation” as its core explanatory framework, focusing on value domains such as marriage, reproduction, gender, and intergenerational obligations. It proposes and operationalizes three measurable value dimensions at the levels of the individual, the couple, and the family: premarital sexual attitudes, gender role beliefs, and conceptions of filial piety. This study examines how these dimensions are associated with contemporary Chinese public attitudes toward homosexuality.

**Method:**

Using data from the Chinese sample of the seventh wave of the World Values Survey (WVS), this study explores how three dimensions of Confucian gender ideology relate to attitudes toward homosexuality. It also examines how gender and gender role beliefs interact with views on filial piety and sexual behavior.

**Result:**

The study finds that in a Confucian cultural context, negative attitudes toward homosexuality are driven not by biological sex, but by the degree of adherence to traditional gender role norms. Among the three dimensions of Confucian gender ideology, more permissive premarital sexual attitudes are positively associated with acceptance of homosexuality, while traditional gender role beliefs and stronger filial piety are significantly negatively associated. Finally, by distinguishing the effects of sex and gender role beliefs, the study shows that gender role beliefs significantly amplify the negative impact of sexual attitudes and filial piety on views toward homosexuality, whereas biological sex has no significant effect.

**Conclusion:**

In the Confucian cultural context, negative evaluations of homosexuality are shaped less by biological sex and more by individuals' adherence to traditional gender norms. Compared to biological identity, homophobic attitudes in Chinese society are more strongly linked to values that uphold the gender order. This study frames Confucian tradition as a key cultural foundation for shaping family ethics and offers a differentiated analysis of family-centered moral values, providing new individual-level empirical evidence for understanding attitudes toward homosexuality in non-Western contexts.

## 1 Introduction

Public attitudes and biases toward homosexuality have undergone a long historical evolution, marked by key shifts including religious and moral disciplining, medical pathologization, and, in recent decades, a rights-based reframing. Cross-cultural studies indicate that varying religious traditions and cultural norms profoundly shape public acceptance of homosexuality through mechanisms of moral authority, communal socialization, and institutional regulation (1, 6).

In many Western societies, early Christian theology regarded same-sex behavior as a sin against the natural order, subjecting homosexuality to prolonged religious and legal condemnation (2, 3). According to traditional Christian theology, individuals are morally obligated to obey the will of God, with the Bible serving as the authoritative expression of that will. Since the Bible prohibits homosexual behavior, it is viewed as a violation of divine will and thus morally wrong (94). A similar mechanism exists in Islamic cultures, where certain verses of the Qur'an are interpreted as condemning same-sex behavior, and many predominantly Muslim countries continue to legally prohibit homosexuality (4, 5). Cross-national surveys also show that societies with higher levels of religiosity tend to exhibit lower levels of acceptance toward homosexuality (6). Since the 19th century, with the rise of psychiatry and sexology, homosexuality gradually shifted from a religious-moral domain to a medicalized one, defined as a diagnosable and treatable “pathological condition,” thereby reinforcing its social stigmatization (7, 8). In the mid-to-late 20th century, propelled by the civil rights and sexual liberation movements, Western societies began to critically reassess traditional norms. The medicalized label was gradually rejected, and homosexuality came to be understood as a variation in sexual orientation rather than a deviant behavior, accompanied by the emergence of anti-homophobia research and equality discourses (9–11). At the same time, gender role beliefs, male gender role stress, authoritarian personality traits, and social dominance orientation have been identified as key theoretical pathways in explaining homophobic attitudes (12, 13). As such, the historical trajectory of attitudes toward homosexuality in the West reflects an overall shift from religious moralization to medical pathologization, and eventually toward a rights- and equality-based framework.

Compared to the developmental trajectory in the West, moral evaluations of sexuality in the Chinese context follow a distinct cultural logic: they are not rooted in monotheistic doctrines, but rather in family ethics deeply shaped by Confucian traditions, emphasizing marriage, reproduction, and intergenerational responsibilities. From a historical perspective, records of same-sex relations in ancient China did not imply institutional endorsement, but were more often context-specific phenomena embedded within a hierarchical family system and ethical order (14). Even during the late imperial era, such practices were merely tacitly tolerated in certain social spaces with low visibility, rather than accepted as part of mainstream values (95). Since the advent of modernity and the introduction of Western sexology and medical knowledge, Chinese society has undergone a transformation from traditional ethical discipline to modern pathological definitions (15). Although medical discourse gradually achieved depathologization after the 1990s, and public attitudes have generally become more tolerant under the influence of globalization (16), the deeply rooted structure of familial responsibilities centered on marriage and reproduction continues to exert strong normative constraints.

Against this backdrop, traditional family values, gender role beliefs, and cultural conservatism not only significantly influence attitudes toward homosexuality, but are also continually reproduced as relationally embedded structures of prejudice within specific social contexts, whose roots often lie not in abstract moral objections to homosexuality, but in the perception that it deviates from expectations tied to family structure and social roles (17–19). This distinction is particularly significant: in contrast to many Western societies where anti-gay prejudice is often framed through religious conceptions of “sin,” prejudice against homosexuality in China more frequently manifests as cultural criticism centered on “unfilial behavior” and the disruption of family normalcy (19, 20). This family-ethics-based logic of regulation, is further reinforced by policy orientations, institutional arrangements, and mainstream public discourse, exhibiting clear characteristics of path dependency. Therefore, the transformation of public attitudes toward homosexuality in China is shaped both by the influence of global rights discourses and by the enduring constraints of Confucian cultural heritage and family-structure logic, forming a distinct sociocultural trajectory that differs from Western patterns of development.

Existing research has confirmed that cultural factors play a significant role in explaining cross-national differences in public attitudes toward homosexuality (92). Studies on Chinese attitudes toward homosexuality have primarily focused on gender role beliefs (21) or family values (19, 22, 23). Among these, the exploration of gender and cultural differences has been one of the central themes. However, the underlying cultural mechanisms remain insufficiently unpacked.

Despite entering the 21st century, Chinese social attitudes remain deeply influenced by Confucian thought. The Confucian emphasis on moral order and collective norms provides a crucial cultural foundation for familist values, and profoundly shapes Chinese perceptions of family, intergenerational relationships, and gender roles (19, 24, 25). Within this cultural framework, the legitimacy of individual behavior is often evaluated based on its contribution to familial obligations and intergenerational continuity (57). Although economic development and transformations in family structure have, to some extent, weakened the overt expression of patriarchal culture (24, 88), existing studies suggest that traditional gender and familial ethics continue to be perpetuated through everyday norms and institutional arrangements (26), and that the influence of traditional culture remains significant (27). Within this framework, the stigmatization of homosexuality is not only a form of exclusion directed at sexual minorities, but also functions to reinforce existing gender role norms. Homosexual relationships are often viewed as deviating from normative standards of heterosexual marriage, family continuity, and gender complementarity within this system, thus serving as a “negative reference point” for the dominant gender order (24).

Accordingly, this study adopts the theoretical perspective of “family-based moral regulation,” and constructs an operationalized explanatory framework centered on the core mechanisms of familial responsibility, reproductive norms, and gender roles to examine public attitudes toward homosexuality. It explores how Chinese public judgments of homosexuality are shaped by the combined influences of reproductive norms, gendered division of labor, and intergenerational obligations. While these values are not equivalent to Confucianism *per se*, the Confucian emphasis on filial piety, familial obligation, and gender hierarchy has historically provided deep cultural support for such family-based ethical structures. This cultural logic is operationalized through three interrelated value dimensions at the levels of the individual, the couple, and the family: sexual norms (attitudes toward premarital sex), gender order (gender role beliefs), and intergenerational obligation (filial piety).

Familial responsibility serves as the ultimate normative reference point; reproductive norms link familial duty to individual sexual attitudes, while gender role beliefs function as a structural pivot: on one hand, they bind marriage and reproduction exclusively to heterosexual relationships; on the other, they reinforce binary gender distinctions, thereby amplifying the perceived “deviance” of homosexuality from the normative family order (17) (Inglehart and Norris, 2003). Crucially, these three dimensions are not parallel or independent variables, but interact within a multi-level structure—family, couple, and individual—to form a synergistic pattern of influence. The analysis draws on data from the seventh wave (2017–2021) of the World Values Survey (WVS), and tests the moderating effects of biological sex and gender role beliefs, in order to more clearly distinguish the key factors shaping attitudes toward homosexuality in the Chinese context.

## 2 Theoretical background and hypotheses

### 2.1 The evolution of attitudes toward homosexuality in the Chinese context: from ethical discipline to medicalized governance

Unlike in many Western societies, where monotheistic religious doctrines have deeply shaped sexual moral regulation, moral regulation of homosexuality in China is less influenced by religion, and cannot be attributed to any single religious or ideological tradition. Instead, it is deeply rooted in a family-based ethical logic centered on marriage, reproduction, and intergenerational obligations. This logic has long been intertwined with Confucian tradition, shaping normative expectations regarding children's marriage, procreation, and gender order, and defining the “legitimacy” of social roles accordingly. As such, it constitutes a foundational framework for evaluating individual sexual practices. Within this ethical structure, historical accounts of same-sex relationships in China should be understood as highly contextualized phenomena embedded in a hierarchical moral order and family-centered institutional arrangements. The existence of such accounts does not necessarily indicate value-based endorsement or institutional legitimacy. Rather, it is more likely that they reflect the limited expressive space afforded to same-sex practices under specific historical conditions (14).

This phenomenon was particularly evident during the late imperial period. At the time, same-sex emotions and relationships were documented in certain texts and social spaces, but such materials primarily reflected interactional patterns within specific social classes or contextual settings, and should not be generalized as indicative of broader societal attitudes. In particular, late Ming and Qing dynasty novels, operas, and other canonical literary works were more closely aligned with elite culture and discursive arenas, with their primary value lying in the presentation of culturally “narratable” resources, rather than directly representing mainstream norms or everyday practices (28). Therefore, the representations in such texts should not be read as evidence of broad value acceptance or widespread tolerance, but rather as instances of low-visibility, context-specific tolerance, or as relatively bounded spaces of expression afforded to certain groups (95).

During the Republican era, Western sexological and medical knowledge gradually entered China, leading to shifts in the ways same-sex desire was understood. Same-sex phenomena increasingly came to be incorporated into a framework of “modern knowledge–classification–governance. Chiang (15) describes this process as an “epistemological modernization, noting that Western sexological concepts were not simply transplanted, but were reassembled within local discourses of science, modernity, and nation-building. Within this discursive framework, some sexological writings began to problematize same-sex desire through a pathologizing lens, with some accounts even proposing heterosexual marriage as a form of “treatment” (15). This shift marked a transition from an ethics-centered understanding to one that emphasized epistemic classification and social problematization.

This medicalized orientation persisted through the Mao era and the early stages of the Reform and Opening-up period. At the time, psychopathic discourse largely inherited the psychiatric framework established during the Republican era. Although the modes of emphasizing family order varied across historical periods, normative expectations surrounding marriage, reproduction, and gender roles remained consistent, thereby significantly constraining the social visibility of homosexuality. During this period, Chinese psychiatric discourse continued to define homosexuality as a specific “personality type,” incorporating it into the global system of psychopathology (90). By the early 1990s, Li (29) through empirical sociological research, pointed out that gay men in China were not absent from social life, but operated under the dual constraints of medicalized discourse and the family institution, forming non-institutionalized social communities in highly concealed ways. She further conceptualized this reality as a structurally constrained subculture, whose continuity did not rely on overt confrontation with mainstream norms, but rather on seeking survival within low-visibility and contextually accommodating spaces (14).

With the development of modern medical discourse, China also experienced a phase of medicalization in its approach to homosexuality. Prior to 1997, sexual activity between men was still classified as the criminal offense of “hooliganism,” and it was not until 2001 that homosexuality was officially removed from the psychiatric diagnostic system (30). Over the past two decades, with the rise of globalization and the internet, discourses of depathologization and sexual rights have entered the public sphere, leading to a certain degree of increased public tolerance (16). Nevertheless, the normative constraints of family responsibility structures—centered on marriage, reproduction, and gender division of labor—remain strong.

This family-structure-based logic of homophobia embeds individual behavior within normative social role expectations related to marriage, reproduction, and lineage continuation, thereby constructing a cultural expectation of “normative intimate relationships.” Within this framework, the perceived “deviance” of homosexuality does not necessarily stem from same-sex behavior itself, but rather from its presumed inability to fulfill obligations of familial continuity and filial piety (19, 31, 32). Although contemporary Chinese society reflects increasing ideological diversity, normative demands concerning family, marriage, and gender order retain significant structural power, a power that has been long intertwined with Confucian cultural tradition (33), continuously shaping public understandings and moral judgments of homosexuality (34, 35).

### 2.2 Gender differences and attitudes toward homosexuality: the cultural embedding of gender norms

Existing research based on Western societies has consistently found stable gender differences in attitudes toward homosexuality, with men generally exhibiting higher levels of prejudice than women (36–39, 92). This gender gap is commonly attributed to men's greater inclination to uphold traditional gender hierarchies, and their stronger adherence to hegemonic masculinity norms. Hegemonic masculinity prescribes that men demonstrate power, dominance, and conformity to heterosexual norms, thereby making men more likely to perceive homosexuality as a threat, and to exhibit higher levels of rejection However, some studies argue that gender differences in attitudes toward homosexuality are not always statistically significant (40, 89).

In Chinese society, the influence of gender differences on attitudes toward homosexuality remains a subject of debate. Some studies have found that men tend to express stronger rejection toward homosexuality than women, and that gender continues to serve as a significant predictor of attitudes toward homosexuality (41, 42, 99). These studies suggest that while acceptance of homosexuality varies across gender groups, men are generally more likely than women to express rejection and negative affect (17). However, other studies have shown that gender differences are not always statistically significant in certain contexts, suggesting that gender may not be a decisive factor influencing attitudes toward homosexuality (22, 43, 91). Underlying this phenomenon, Confucian gender and family norms may offer critical explanatory insights.

Within the familist value structure rooted in Confucian tradition, a man is recognized as such insofar as he fulfills the familial responsibilities and obligations expected of his gender role. If a biological male remains unmarried and fails to continue the family lineage, his masculinity may be questioned, and he may not be regarded as a “real man” in the full cultural sense (44, 45). The same applies to women: if they fail to bear a male heir for their husband's family (35), they may be seen as falling short of being a “qualified” wife. Consequently, homosexual individuals who do not conform to traditional gender expectations or fulfill familial roles often face heightened social scrutiny and cultural rejection. Based on the above analysis, the following hypothesis is proposed:

**H1**: Compared to women, men hold more negative attitudes toward homosexuality.

### 2.3 The role of gender role beliefs and heteronormative norms in shaping attitudes toward homosexuality

Gender ideology, as a cultural construct, is deeply intertwined with individual attitudes toward homosexuality. Mead (46) argued that the formation of gender is largely shaped by culture rather than biological sex, and that gender differences in temperament and behavioral norms are rooted in cultural rather than biological distinctions. As gender role beliefs reflect culturally embedded ideologies, they are considered key to understanding cross-cultural differences in attitudes toward homosexuality (92). Numerous studies have demonstrated the negative influence of gender role traits on prejudice against homosexuality. For instance, the “gender inversion hypothesis” suggests that homosexual individuals are often perceived as crossing gender boundaries: gay men are viewed as “feminine,” while lesbian women are seen as “masculine” (47, 48). It is precisely these gender-nonconforming behaviors—feminized traits in gay men and masculinized traits in lesbian women—that create cognitive dissonance in gender perceptions, challenging conventional understandings of traditional gender roles and triggering socially defensive moral judgments (19, 49).

In the Chinese context, this mechanism is reinforced by deeper cultural structures. Traditional Confucian ethics emphasize a gendered order of “inner vs. outer domains” (50), establishing male dominance in both public and domestic spheres through gendered role differentiation, while women are confined to the private realm, responsible for caregiving and maintaining familial morality (51, 93). This gender structure constitutes not only a functional division of labor but also a hierarchical order. In historical China, men were assigned an idealized trajectory of “cultivating the self, regulating the family, governing the state, and bringing peace to the world,” with their social achievement predicated on the establishment of authoritative status within the family. Although women played important roles within the family sphere, they lacked substantive power and societal recognition (51, 96). This structural inequality has continuously reproduced the cultural norm of “male superiority and female inferiority,” reinforcing the rejection and fear of non-traditional gender expressions (52).

Within this cultural context, heterosexual relationships are often regarded as the only “legitimate” form of intimacy, as they conform to the logic of family continuity and gender complementarity. Same-sex relationships and marriages are viewed as violations of traditional gender roles, and as posing potential challenges to family structure and social stability (17, 53). Consequently, traditional gender role beliefs not only reflect culturally prescribed expectations regarding heterosexual relationships, but also serve as a crucial basis for evaluating the legitimacy of same-sex relationships. Based on the above analysis, the following hypothesis is proposed:

**H2:** The stronger an individual's traditional gender role beliefs, the more negative their attitudes toward homosexuality.

### 2.4 Filial piety and intergenerational obligation: familist moral regulation in the context of confucian culture

For thousands of years, filial piety has remained a core tenet of Confucian culture. In Chinese culture, filial piety is highly institutionalized and carries strong moral authority. Its uniqueness lies in the fact that it is not merely an emotional expression of respect for older adults, but a comprehensive ethical system that governs individual behavior. Weber (54) emphasized the centrality of filial piety in Chinese culture, dentifying it as a paradigmatic virtue that sustains Confucian ethics and social norms. He argued that filial piety constitutes the foundation of moral values and social organization, serving as the source from which all other virtues are generated and cultivated. The intergenerational reciprocity inherent in filial piety has long functioned as a core social bond in Chinese society, giving rise to a cultural expectation whereby parents feel obligated to care for their children, while adult children are expected to support their aging parents and fulfill their wishes (19).

Although the ethical responsibility of intergenerational caregiving is not unique to East Asian societies—for instance, filial values have also been positively associated with caregiving behaviors in Muslim communities in South Africa and France (55, 56)—filial piety in Confucian culture occupies a particularly distinctive position. Compared to Western societies, which emphasize individual autonomy and generational equality, filial piety in China is not merely an emotional expression of respect, but an institutionalized and unconditional moral obligation Its core components include three dimensions: “respect,” “nurture,” and “continuity”: “Respect” requires individuals to maintain reverence for their elders at a spiritual level, demonstrating achievements that make parents proud. “Nurture” prescribes the responsibility of material reciprocation and long-term caregiving; “Continuity” emphasizes the continuation of the family bloodline (57, 97). This moral framework constructs a family-centered social order—wherein an individual's moral worth, social identity, and even life choices are often oriented around the needs and collective interests of the family.

More crucially, in Confucian culture, respect for parents and the continuation of the patrilineal bloodline are regarded as fundamental prerequisites for social harmony and effective governance. Within this cultural framework, filial piety is not only a private moral virtue, but also functions as a key mechanism for maintaining hierarchical social structures and political order (54). The continuation of the patrilineal bloodline forms the core logic of the Chinese kinship system. As Mencius famously stated, “There are three forms of unfilial conduct, and having no descendants is the most severe,” this classic assertion established reproduction as a central filial duty, thereby granting heterosexual marriage an unshakable institutional legitimacy (93). As every “normal” man and woman is expected to marry a partner of the opposite sex and have children, parents likewise hope that their children will “properly” fulfill their gendered roles. The authoritative dimension of filial piety thus profoundly shapes sexual norms, particularly attitudes toward homosexuality, as forming a traditional family and continuing the family line are viewed as among the most essential filial obligations in traditional Chinese culture (19). Meeting parental expectations is a key component of filial duty. Heterosexual marriage derives its legitimacy through this process of intergenerational transmission within the family. In light of the above discussion on filial piety, the following hypothesis is proposed:

**H3:** Stronger endorsement of filial piety is associated with more negative attitudes toward homosexuality.

### 2.5 Sexual attitudes and premarital sexual norms within the confucian cultural framework

Within the constraints of the traditional Confucian ethical system, sexual behavior has always been strictly regulated under the framework of *li* (ritual propriety). Sexual activity is not merely regarded as a private act of physical desire, but is also seen as serving collective functions, such as ensuring lineage continuity, maintaining clan order, and contributing to broader social stability (52, 58). Within this moral framework, only sexual activity that takes place within heterosexual marriage is considered legitimate and consistent with *li*. As a result, premarital sex is viewed as a violation of norms concerning female chastity and familial discipline, and as an affront to the moral and hierarchical order of the family and lineage (98, 100).

The highly codified regulation of sexual behavior in Confucian culture historically led to extremely strict constraints on premarital sexual activity. In traditional society, both premarital sexual activity and romantic relationships before marriage were socially prohibited. Marriages were arranged strictly under parental authority. Behaviors such as premarital dating, the exchange of tokens, and physical contact were regarded as immoral and indicative of a lack of chastity. Marriage, in the Confucian sense, was perceived as an act of loyalty to one's family, rather than a union based on personal affection or romantic love (96, 100). Even in contemporary China, the cultural inertia of conservative sexual attitudes remains evident. A national survey found that in early 1990s China, 89.2% of premarital sexual activity occurred only between individuals and their future spouses (59). Moreover, compared to Western societies, Chinese individuals show generally lower tolerance for premarital and extramarital sex, reflecting a broadly conservative sexual outlook (60).

In Confucian ethics, the moral disapproval of premarital sex is not merely directed at individual conduct, but reflects the strict regulation of sexual behavior through *li* (ritual propriety) and *jiafa* (family rules). In contemporary research, such patterns are typically classified under the broader concept of “sexual moral conservatism,” which refers to the moral framing of all sexual acts outside the bounds of marriage, reproduction, and familial order as deviant (61). Confucian sexual ethics can be seen as the culturally specific embodiment of this moral orientation within the Chinese context. Within this conservative framework, premarital sex and homosexuality are no longer treated as isolated categories of behavior, but are incorporated into the same normative system as forms of “boundary-crossing” sexual conduct. Both are viewed as challenges to the traditional marriage–reproduction–lineage continuity order, and thus elicit similar forms of moral rejection (62). Based on this reasoning, the following hypothesis is proposed:

**H4:** Individuals with more conservative sexual attitudes are more likely to hold negative views toward homosexuality.

### 2.6 Gender differences as a moderator of cultural norms and sexual attitudes

As previously discussed, in Confucian culture, marriage, and sexual behavior are not merely matters of individual choice, but are closely tied to morally sanctioned obligations surrounding family lineage continuation. Especially within the patriarchal family structure, men are typically regarded as the carriers of the patrilineal bloodline, and are therefore subject to heightened marital and reproductive responsibilities and cultural expectations (19, 63, 93). Within this value framework, men, owing to their central role in the kinship system, are often assigned greater obligations and expectations related to marriage and reproduction.

Unlike women, who are regulated by norms of chastity in traditional culture, men are regulated within a moral framework that emphasizes their responsibility to continue the family lineage (64). Existing studies have noted that sexual minorities in China commonly face marital pressure from both parents and society, and such pressure is recognized as one of the key sources of social stress linked to sexual orientation (65). This pressure to marry and reproduce is often more culturally pronounced for male individuals (66). Men are typically seen as the bearers of family honor and the continuation of ancestral lineage, serving as the essential link to maintaining the family line. Compared to women, men who remain unmarried, childless, or fail to continue the family line are more likely to be perceived as “failing in their duties” or “unfilial.” Male homosexuals, whose sexual practices typically do not involve heterosexual marriage or reproduction, are thus more prone to conflict with traditional family norms (29). Therefore, within strong cultural and moral frameworks, male individuals—due to their patrilineal obligations and gendered expectations—may exhibit stronger negative attitudes toward homosexuality. Based on this, two hypotheses concerning interaction effects involving gender are proposed:

**H5a:** Among individuals with conservative sexual attitudes, men are more likely than women to hold negative attitudes toward homosexuals.

**H5b:** Among individuals with strong filial piety beliefs, men are more likely than women to hold negative attitudes toward homosexuality.

### 2.7 Gender role beliefs as a moderator of normative pressures on homosexuality

Beyond biological sex, individuals' endorsement of traditional gender role beliefs may also significantly influence how cultural values shape their attitudes toward homosexuality. In Western societies, gender role norms are often embedded in religious doctrines or individualistic gender expectations, whereas in Confucian culture, gender division of labor is not only a social arrangement, but also closely tied to family ethics, intergenerational obligations, and moral duties of patrilineal continuity, thereby forming a more stringent cultural disciplinary mechanism against non-traditional gender expressions (50). Traditional cultural norms draw clear distinctions between the expected social roles of men and women: men are expected to take on economic and authoritative roles in the public sphere, while women are positioned within the domestic sphere, bearing responsibilities for reproduction and moral maintenance (67). These gender divisions not only shape behavioral expectations, but also inform cultural understandings of the purpose and meaning of sexual behavior. Individuals who strongly endorse traditional gender roles, tend to support stricter sexual norms, emphasizing that sex should occur within marriage and serve reproduction and gender complementarity (68). Heterosexual relationships, as they conform to the gender logic of “men in public, women at home,” are typically viewed as natural and legitimate, whereas homosexual relationships are seen as violating this gender order (69). Prior studies on gender role beliefs suggest that gay men experience greater cultural pressure than lesbian women (70, 71). A study in China further found that male sexual minorities report stronger senses of family obligation than their female counterparts, suggesting that men in China may face greater pressure to marry and continue the family line. Based on this, two hypotheses involving interaction effects with gender role beliefs are proposed:

**H6a:** Traditional gender role beliefs amplify the negative association between conservative sexual attitudes and attitudes toward homosexuals.

**H6b:** Traditional gender role beliefs amplify the negative association between filial piety beliefs and attitudes toward homosexuality.

## 3 Method

### 3.1 Data

This study utilized data from the seventh wave of the World Values Survey (WVS), conducted between 2017 and 2021. The WVS is a global social science research network led by an international team of scholars, dedicated to examining changes in social values and their impact on social and political life. The survey was administered anonymously to fully protect respondents' privacy.

The dataset included a total of 94,278 respondents, of which 3,036 were from mainland China. Samples with invalid responses (including “don't know,” “no answer/refused,” “not applicable,” or “missing/other reasons for inapplicability”) were excluded. Ultimately, this study obtained 2,535 valid samples, including 1,140 male respondents (44.97%) and 1,395 female respondents (55.03%).

### 3.2 Measure

Dependent Variable. The dependent variable is based on an ordinal-scaled item designed to measure respondents' attitudes toward homosexuality. The survey question asks: “Do you think homosexuality is always acceptable, never acceptable, or something in between?” Respondents answered using a 10-point Likert scale (1 = absolutely unacceptable, 10 = absolutely acceptable). This measure reflects a general normative attitude, capturing the overall level of acceptance an individual holds toward homosexuality. Higher scores indicate greater acceptance of homosexuality (Table 1).

**Table 1:** Sample characteristics.

**Variables**	**Definitions**	***N* (%)**	**Mean (SD)**	**Min/Max**
Acceptance homosexuality	1 = Never Justifiable 10 = Always Justifiable		2.39 (2.49)	1/10
Gender	1 = female, 0 = male	1,395 (55.03%) 1,140 (44.97%)		
Acceptance premarital sex	1 = Never Justifiable 10 = Always Justifiable		3.83 (2.98)	1/10
Traditional gender role beliefs	1 = traditional gender role 0 = modern gender role	1,492 (58.86%) 1,043 (41.14%)		
Filial piety	1 = Strongly disagree 4 = Strongly agree		3.48(0.71)	1/4
Marital status	1 = married, 0 = single	1,235 (88.53%) 160 (11.47%)		
Age group	Group 1 = 18–35 Group 2 = 36–49 Group 3 = 50–70	833 (32.86%) 874 (34.48%) 828 (32.66%)		1/3
Education	1 = Early childhood education/no education 2 = Primary education 3 = secondary education 4 = Bachelor or equivalent 5 = Master or equivalent and above	137 (5.4%) 410 (16.17%) 1,376 (54.28%) 585 (23.08%) 27 (1.07%)		1/5
Subjective social status	1 = Lower class 2 = Working class 3 = Lower middle class 4 = Upper middle class 5 = Upper class	460 (18.15%) 916 (36.13%) 1,089 (42.96%) 65 (2.56%) 5 (0.2%)		1/5

Independent Variables. This study focuses on three core value-related independent variables: attitudes toward premarital sex, gender role beliefs, and filial piety. Although these orientations are often examined within the framework of Confucian cultural traditions, this study does not treat them as direct reflections of Confucianism, but rather as contemporary manifestations of traditional family ethics. (1) Gender. Gender is coded as a binary variable, with male = 0 and female = 1.

(2) Acceptance Premarital Sex. This variable is measured using the following question: “Do you think premarital sex is always acceptable, never acceptable, or somewhere in between?” Respondents answer on a 1–10 point Likert scale (1 = absolutely unacceptable, 10 = absolutely acceptable). In the analysis, this variable is reverse-coded, with higher scores indicating less tolerance toward premarital sex.

(3) Gender Role Beliefs. Gender role beliefs are measured using three items, where respondents indicate their level of agreement with the following statements: “In general, men are better suited than women to be political leaders,” “A university education is more important for a boy than for a girl,” “In general, men are better suited than women to be top business executives.” (Responses are coded on a 4-point scale: 1 = strongly agree, 4 = strongly disagree). To identify individuals who strongly endorse traditional gender norms, responses are first reverse-coded, and then transformed into a binary variable: 0 = modern gender role orientation, 1 = traditional gender role orientation. The binary coding is not intended to capture attitudinal strength, but rather to distinguish the directional orientation of normative beliefs, which aligns with prior studies that adopt similar strategies for analytical clarity (1, 72). Admittedly, this approach simplifies the continuity and domain-specific variations of gender ideology to some extent, but it helps to highlight the directional role of gender norm endorsement in shaping attitudes toward homosexuality. Moreover, the binary variable facilitates comparative analysis with the biological sex variable, allowing for a clearer distinction between the effects of gender identity and gender ideology on attitudinal outcomes.

(4) Filial Piety. This study conceptualizes filial piety as a proxy indicator reflecting individuals' orientation toward family responsibility and intergenerational obligation. Due to limitations in secondary data availability, three items were selected for operationalization, aiming to capture the Confucian ethical logic of “respect, nurture, and continuity”: “One of my main goals in life is to make my parents proud of me,” “Adult children have a duty to provide long-term care for their parents,” “Having children is a responsibility to society.” This construct is intended to measure family obligation orientations related to filial piety, though it does not comprehensively capture all dimensions of the concept. Respondents rated their level of agreement with each statement, and all items were reverse-coded in the analysis to ensure consistent directionality. Higher scores indicate stronger endorsement of filial piety beliefs.

Control Variables: This study controls for a set of sociodemographic factors, including marital status, age, educational attainment, and subjective social class. Marital status was categorized into two groups: unmarried and married, with the married category including married, cohabiting, divorced, separated, or widowed individuals. Educational attainment was coded on a scale from 1 to 5: 1 = pre-primary education (ISCED 0) or no formal education, 5 = master's degree or equivalent and above (ISCED 7 and 8). Subjective social class was measured using a five-point scale and reverse-coded, with 1 representing the lower class and 5 representing the upper class.

## 4 Result

Table 2 presents the means and standard deviations of respondents' attitudes toward homosexuality, attitudes toward premarital sex, gender role beliefs, and endorsement of filial piety, along with comparisons of these variables by gender. Overall, the sample exhibited relatively negative attitudes toward homosexuality (Mean = 2.39, SD = 2.49), conservative attitudes toward premarital sex (Mean = 3.82, SD = 2.97), a tendency toward traditional gender role beliefs (Mean = 0.59, SD = 0.49), and strong endorsement of filial piety (Mean = 2.94, SD = 0.74).

**Table 2:** Mean value of dependent and independent variables with difference between genders.

**Variables**	Full sample (***n*** = 2,535)		Male (***n*** = 1,140)		Female (***n*** = 1,395)		**Difference between male and female**	**F/P**
	**Mean**	**SD**	**Mean**	**SD**	**Mean**	**SD**		
Acceptance homosexuality	2.39	2.49	2.32	2.40	2.44	2.56	−0.12	1.48/0.225
Acceptance premarital sex	3.82	2.97	4.04	2.99	3.65	2.95	0.39^***^	10.77/0.001
Traditional gender role beliefs	0.59	0.49	0.63	0.48	0.55	0.50	0.08^***^	15.92/0.000
Filial piety	2.48	0.71	2.50	0.70	2.47	0.71	0.04	1.92/0.166

^*^p < 0.1, ^**^p < 0.05, ^***^p < 0.01.

Mean comparison tests found no significant gender differences in attitudes toward homosexuality or endorsement of filial piety. Men were more inclined toward traditional gender role beliefs than women (men: Mean = 0.63, SD = 0.48; women: Mean = 0.55, SD = 0.50), but displayed more permissive attitudes toward premarital sex (men: Mean = 4.04, SD = 2.99; women: Mean = 3.65, SD = 2.95).

Table 3 presents the results of the regression analysis. Model 1 indicates that, after controlling for demographic variables such as marital status, age, educational attainment, and subjective social class, women, single individuals, and younger respondents exhibit higher acceptance of homosexuality. The adjusted R-squared of Model 1, which includes only the control variables, is relatively low. Hypothesis H1 is supported.

**Table 3:** Ordinary least squares regression toward attitudes toward homosexuality.

**Variables**	**Model 1**	**Model 2**	**Model 3**	**Model 4**
Female	0.202^**^	0.244^***^	0.119	0.239^***^
	(0.10)	(0.09)	(0.50)	(0.09)
Acceptance premarital sex		0.296^***^	0.278^***^	0.358^***^
		(0.02)	(0.02)	(0.02)
Traditional gender role beliefs		−0.293^***^	−0.161	−0.647
		(0.09)	(0.13)	(0.49)
Filial piety		−0.469^***^	−0.490^***^	−0.584^***^
		(0.06)	(0.10)	(0.09)
Marital status	−0.924^***^	−0.835^***^	−0.839^***^	−0.810^***^
	(0.15)	(0.14)	(0.14)	(0.141)
Age group	−0.400^***^	−0.165^**^	−0.161^**^	−0.164^**^
	(0.07)	(0.07)	(0.07)	(0.06)
Education	0.258^***^	0.023	0.052	0.063
	(0.07)	(0.06)	(0.06)	(0.06)
Subjective social status	0.185^***^	0.179^***^	0.183^***^	0.177^***^
	(0.06)	(0.06)	(0.06)	(0.06)
Gender^*^Acceptance premarital sex			0.033	
			(0.03)	
Gender^*^Traditional gender role beliefs			−0.235	
			(0.18)	
Gender^*^filial piety			0.039	
			(0.13)	
Traditional gender role ^*^Acceptance premarital sex				−0.106^***^
				(0.03)
Traditional Gender role ^*^filial piety				0.222^*^
				(0.13)
_cons	2.670^***^	3.386^***^	3.451^***^	3.492^***^
	(0.33)	(0.38)	(0.47)	(0.46)
*N*	2,535	2,535	2,535	2,535
Adjusted R-square	0.080	0.239	0.240	0.242

^*^p < 0.1, ^**^p < 0.05, ^***^p < 0.01.

Model 2 shows that among the three Confucian values, a permissive attitude toward premarital sex is positively associated with acceptance of homosexuality (β = 0.296, *p* < 0.01), whereas filial piety (β = −0.469, *p* < 0.01) and traditional gender role beliefs (β = −0.293, *p* < 0.01) are negatively associated with acceptance of homosexuality. The R-squared of Model 2 increases to 0.239, indicating stronger explanatory power compared with Model 1. This suggests that the three Confucian cultural factors play a significant role in shaping Chinese attitudes toward homosexuality. Hypotheses H2, H3, and H4 are supported.

Model 3 tested the moderating effect of gender. The results show that the interaction terms between gender and the three cultural dimension variables—attitudes toward premarital sex (β = 0.278, SE = 0.02, *p* < 0.01), gender role beliefs (β = −0.161, SE = 0.13, *p* = 0.231), and filial piety (β = −0.490, SE = 0.10, *p* < 0.01)—are all non-significant. This indicates that gender does not significantly moderate the effect of Confucian cultural values on attitudes toward homosexuality. Therefore, hypotheses H5a and H5b are not supported.

**Figure 1:**
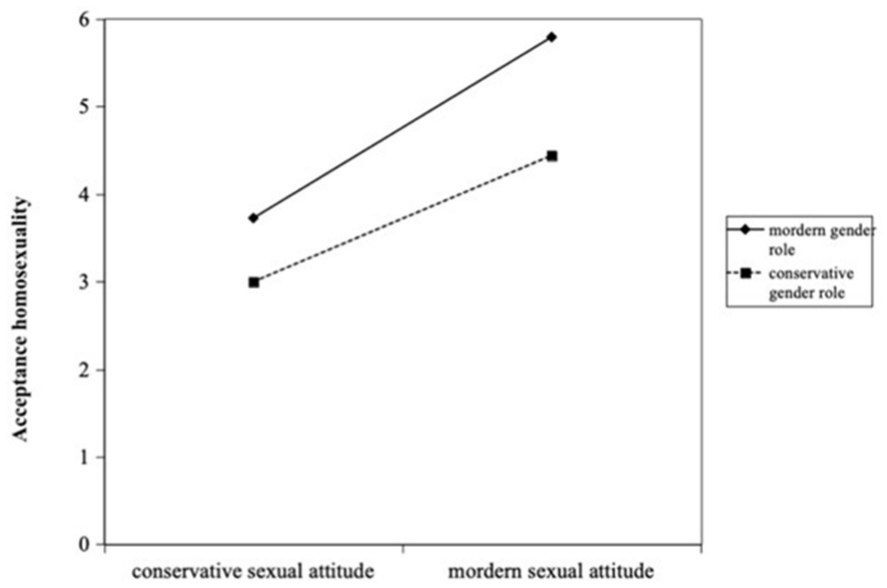
The moderating effect of traditional gender role beliefs in acceptance premarital sex and acceptance homosexuality.

**Figure 2:**
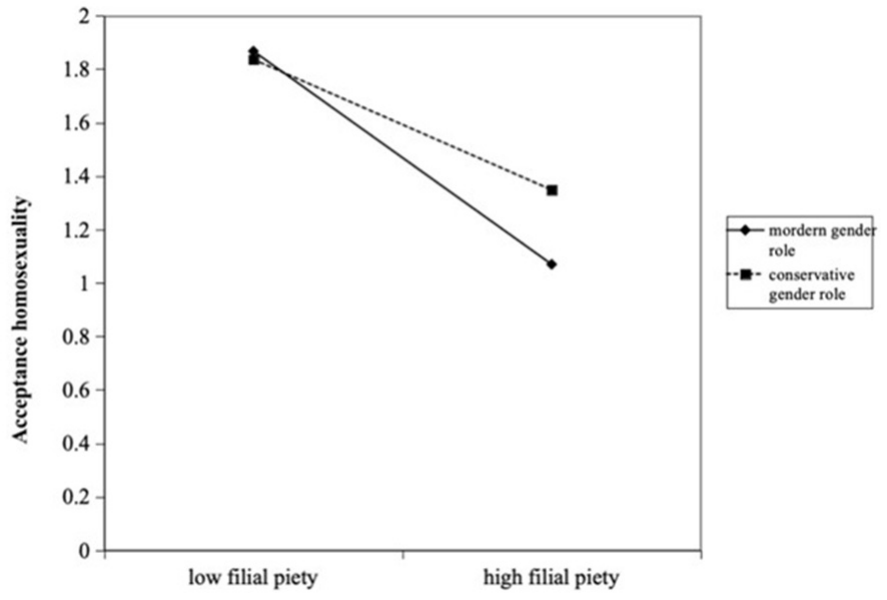
The moderating effect of traditional gender role beliefs in filial piety and acceptance homosexuality.

Model 4 tested the moderating effect of traditional gender role beliefs. The interaction between traditional gender role beliefs and attitudes toward premarital sex (β = −0.106, SE = 0.03, *p* < 0.01) is significantly negative, indicating that traditional gender role beliefs reduce the positive impact of permissive sexual attitudes on acceptance of homosexuality. Additionally, the interaction between traditional gender role beliefs and filial piety (β = 0.222, SE = 0.13, *p* < 0.01) is significantly positive, indicating that traditional gender role beliefs amplify the negative effect of filial piety on acceptance of homosexuality. Figures 1, 2 illustrate the decomposition of the moderating effects of gender role beliefs on the influence of attitudes toward premarital sex and filial piety. Hypotheses H6a and H6b are supported.

Figure 1 illustrates the moderating role of gender role beliefs on the relationship between sexual attitudes and attitudes toward homosexuality: regardless of whether gender role beliefs are conservative or modern, more permissive sexual attitudes increase acceptance of homosexuality. The positive effect is stronger for individuals with modern gender role beliefs and weaker for those with traditional beliefs.

Figure 2 illustrates the moderating effect of gender role beliefs on the relationship between filial piety and attitudes toward homosexuality. Overall, regardless of the level of gender role beliefs, higher levels of filial piety are associated with more negative evaluations of homosexuality. The negative impact is greater among individuals with modern gender role beliefs.

## 5 Discussion

As one of the most influential philosophical systems in Chinese history, Confucianism has played a profound and enduring role in shaping individual values, particularly leaving a lasting imprint on gender norms, reproductive duties, and intergenerational responsibilities. In this sense, Confucian values not only form the core moral foundation of intimate relationships and family structures in Chinese society, but also provide a cultural context for understanding the moral evaluations of intimate relationships within Chinese society, in which non-traditional intimate relationships (especially homosexuality) are more likely to be subjected to normative discussions (1, 41).

In the family-centered cultural framework of China, intimate relationships are often viewed as a social obligation rather than purely an individual choice. This ethical structure is typically understood as being intertwined with gender order, reproductive norms, and intergenerational responsibilities, and may influence individuals' moral evaluations of intimate relationships through long-term socialization processes. In contrast, Western societies emphasize individual autonomy and the freedom of choice, and their individualistic analytical frameworks may have limitations when applied to understanding the norms of intimate relationships in the Chinese context, especially when explaining the relationship between sex, marriage, and reproductive responsibilities.

In light of these cultural characteristics, this study focuses primarily on several measurable value orientations at the individual level, without making direct causal inferences about macro-level cultural mechanisms. Specifically, the analysis centers on three highly structured and concrete cultural value dimensions: attitudes toward premarital sex, gender role beliefs, and filial piety. These dimensions can be understood as individual-level normative cognitions linked to traditional cultural frameworks, corresponding to three core themes: sexual-marital order, gender division of labor, and familial continuity obligations.

Attitudes toward premarital sex reflect individuals' endorsement of the cultural proposition that sexuality should be confined within legal marriage and serve the purpose of familial continuity. Filial piety beliefs capture the ethical responsibility associated with heterosexual marriage and reproductive obligations. Gender role beliefs function as a cross-cutting normative structure, moderating the relationships between various value orientations and attitudinal outcomes. Empirical analysis reveals that individuals who more strongly endorse gender hierarchy, are more likely to perceive premarital sex and same-sex relationships—given their lack of traditional reproductive function—as dual violations of familial responsibility and gender norms, thereby expressing more negative evaluations.

Therefore, this study argues that attitudes toward homosexuality in Chinese society should be understood as the outcome of multiple interrelated individual-level value orientations, rather than the direct product of a single ideological system. The combination of these values, within a cultural context dominated by Confucian family ethics, is more likely to be associated with normative labels such as “non-marital” and “non-reproductive.” In addition, we further examined the moderating effect of gender role beliefs, exploring whether it amplifies the effects of other cultural value pathways, with the aim of better understanding how cultural values interactively shape the social evaluation of sexual minority groups.

### 5.1 Sexual moral conservatism and evaluative orientations toward homosexuality

This study finds that conservative attitudes toward premarital sex are significantly associated with negative evaluations of homosexuality among Chinese respondents. This relationship can be interpreted as a form of attitudinal consistency within a cultural context dominated by familism ethics, wherein individuals tend to associate sexual behavior with family responsibilities and intergenerational obligations. Previous studies have pointed out that within family-centered moral discourse, sexual behavior is normatively confined to the institution of marriage and closely tied to reproductive responsibility, and behaviors that deviate from this norm are often framed as potential threats to family order in such cultural contexts (73). Therefore, individuals with conservative orientations are more likely to endorse a sexuality moral framework centered on marriage and reproduction, and to express stronger disapproval of intimate relationships that do not conform to this structure.

Relevant studies have shown that within a family-centered value system, premarital chastity is not only viewed as a marker of individual moral integrity, but is also culturally framed as a means to protect family reputation and ensure the purity of patrilineal lineage (74). Within this normative understanding—where sexuality, marriage, and reproduction are closely intertwined—same-sex relationships, lacking traditional reproductive functions, are more readily associated with deviations from established marital and reproductive norms, particularly among individuals with a strong endorsement of conventional sexual norms, such perceived deviations are more likely to be moralized and judged as “unacceptable.”

Moreover, in contemporary Chinese public discourse, marriage and childbearing continue to be widely regarded as symbols of adulthood and social responsibility (75, 76). Against this backdrop, individuals' conservative attitudes toward premarital sex may be interpreted as ethically grounded evaluations of the marriage system and familial obligations. The interconnection among these value orientations provides a critical individual-level perspective for understanding variation in attitudes toward homosexuality.

### 5.2 Structural differences in attitudes toward homosexuality by gender role beliefs

This study finds that traditional gender role beliefs are significantly associated with individuals' negative attitudes toward homosexuality. Such beliefs typically associate men and women with distinct gender norms—for example, men are expected to embody strength, control, and dominance in the public sphere, while women are expected to exhibit traits such as gentleness, submissiveness, and domestic orientation (77, 78). In the Chinese context, such gender role assumptions are often closely tied to normative discourses surrounding family and reproduction, and reflect strong societal expectations.

Existing research indicates that within normative frameworks emphasizing traditional gender role divisions, heterosexual marriage is more likely to be perceived as the appropriate form of intimacy aligned with gender expectations, whereas same-sex relationships are more often interpreted as deviations from the established gender order (79). In particular, the egalitarian role structure observed in male same-sex relationships, and female same-sex relationships' departure from traditional heterosexual dependency models, are frequently viewed as inconsistent with prevailing gender norms (52).

In this context, individuals who strongly endorse the gender division of labor—where men dominate the public sphere and women are responsible for the domestic sphere—are more likely to perceive same-sex relationships as inconsistent with their gendered expectations of intimacy, and consequently exhibit more negative attitudes toward homosexuality. In other words, traditional gender role beliefs are closely linked to individuals' evaluations of intimate relationships, and this association offers important insights into the variations in attitudes toward homosexuality in Chinese society.

### 5.3 Filial piety and attitudes toward homosexuality: a family responsibility perspective

The findings of this study indicate a significant association between individuals' endorsement of filial piety and their negative attitudes toward homosexuality. As a value orientation that emphasizes intergenerational responsibility and familial obligation, filial piety provides a crucial cultural reference point for understanding moral evaluations of intimate relationships in Chinese society. Previous studies have suggested that within cultural contexts that stress family continuity and intergenerational duties, marriage choices are often not merely viewed as personal emotional decisions, but are also closely tied to expectations of fulfilling familial responsibilities (80, 81).

In family-centered social contexts, marriage and procreation are often symbolically associated with family continuity and the fulfillment of parental expectations (82, 83). Within this cultural framework, heterosexual marriage—given its close alignment with reproductive practices—is more readily viewed as a filial and socially legitimate path within the family system. In contrast, same-sex relationships, lacking the reproductive capacity embedded in traditional norms, are often excluded from normative discourses surrounding “familial responsibility,” thereby becoming more morally contested in evaluative judgments.

Related studies have further suggested that filial piety endorsement is closely associated with the degree to which individuals value parental opinions and family norms (84). Against this backdrop, individuals with stronger filial piety beliefs are more likely to prioritize familial responsibilities and intergenerational obligations when evaluating intimate relationships. Thus, the association between filial piety and attitudes toward homosexuality may be interpreted as a value-consistency pattern linking familial responsibility orientations to normative assessments of intimate relationships.

### 5.4 Gender role beliefs as a moderator of value–attitude associations

The findings show that none of the three interaction terms involving biological sex reached statistical significance, suggesting that biological sex does not moderate the relationship between Confucian-related value orientations and attitudes toward homosexuality. This indicates that biological sex itself may not be a decisive factor in shaping attitudes toward homosexuality. In contrast, individuals' endorsement of traditional gender role norms—namely, gender role beliefs—appears to be a more meaningful predictor of variations in attitudes toward homosexuality.

The results indicate that traditional gender role beliefs significantly strengthen the association between conservative attitudes and negative views toward homosexuality. This finding suggests that although gender role beliefs are closely related to biological sex, they are better understood as a cultural value orientation that reflects individuals' endorsement of the legitimacy of gender hierarchy and social division of labor (85, 86). Therefore, in explaining differences in attitudes toward homosexuality, subjective adherence to gender norms may be more predictive than biological sex itself. Along the premarital sex pathway, the moderating effect of gender role beliefs suggests that, when individuals strongly endorse a traditional marriage–procreation framework, the link between sexual conservatism and negative attitudes toward homosexuality becomes more pronounced. Specifically, among individuals who strongly identify with hierarchical gender norms, sexual behavior is more likely to be interpreted through the lens of familial obligation and social morality. When individuals believe that sex should occur strictly within heterosexual marriage and serve the purpose of family continuity and gender-based division of roles, same-sex relationships that deviate from this structure are more likely to be perceived as “non-normative,” thereby triggering negative emotions and moral disapproval.

Along the filial piety pathway, the moderating effect of gender role beliefs is relatively weak but still observable. For individuals who strongly endorse traditional gender hierarchies, filial piety is more likely to be linked with expectations of heterosexual marriage and procreation. As a result, same-sex relationships that deviate from these marital and reproductive norms are more likely to elicit negative evaluations.

Overall, gender role beliefs reflect individuals' cultural endorsement of the societal gender order, and serve to amplify the associations between various value orientations and attitudes toward homosexuality. Compared to biological sex as an identity marker, gender role beliefs offer a stronger explanation for individual differences in attitudes toward homosexuality.

## 6 Contribution and limitations

This study expands the analytical perspective on attitudes toward homosexuality in the Chinese context, both theoretically and empirically. Unlike studies that treat “Confucian culture” as a singular macro-level explanatory framework, this research defines it as a deep historical and cultural backdrop, and operationalizes its embedded family- and gender-related values into three measurable and identifiable dimensions at the individual level. The study is framed around the value linkages of “individual–gender–family,” focusing on gender relations (gender role beliefs), intergenerational relations (filial piety), and attitudes toward sexual behavior (views on premarital sex), which are operationalized for measurement. The findings reveal an ordering of main effects across the three dimensions: sexual attitudes, gender relations, and intergenerational relations, thus demonstrating how family-oriented moral values systematically relate to attitudes toward homosexuality across various levels. This provides a practical analytical framework for future research on the individual-level mechanisms underlying such associations.

Secondly, the study distinguishes between the effects of biological sex and gender role beliefs, finding that gender role beliefs significantly amplify the negative effects of sexual attitudes and filial piety on attitudes toward homosexuality. Although women generally exhibit greater acceptance, it is not biological sex *per se* that strengthens the negative associations between sexual attitudes, filial piety, and attitudes toward homosexuality, but rather the extent to which individuals endorse traditional gender norms. ideological differences in gender, rather than mere sex differences, are more effective in explaining variations in attitudes toward homosexuality in the Chinese context. This finding shifts the analytical focus from “gender differences” to “gender ideological differences,” offering a more nuanced and structured explanation of homophobic attitudes in Chinese society.

Despite these contributions, this study has several limitations. First, since the dependent variable reflects an overall normative attitude, the analysis primarily aims to uncover the relationship between family-related value orientations and attitudes toward homosexuality, rather than distinguishing between different levels of acceptance mechanisms.Additionally, the study relies on cross-sectional data, meaning that it cannot capture the causal effects of how cultural dimensions evolve over time in shaping attitudes, thus limiting the ability to make robust causal inferences.

Secondly, some key constructs rely on proxy indicators from survey data, and certain dimensions are measured using single or limited items, which somewhat limits the ability to capture the internal complexity and multidimensional structure of cultural concepts. For example, attitudes toward premarital sex and filial piety endorsement empirically reflect family responsibility and moral evaluation orientations, but cannot be equated with a comprehensive measurement of Confucian thought or its ethical system. Similarly, the measurement of gender role beliefs also has certain limitations. The indicators selected in this study primarily capture individuals' gender ideological tendencies along the “traditional–nontraditional” dimension, While this binary approach helps identify general normative adherence, it inevitably compresses the subtle variations in attitude distribution, thus limiting the depiction of the continuous variations within gender beliefs. Therefore, this indicator should be regarded as an approximate measure of “traditional gender norm adherence,” rather than a complete reflection of its complexity or intensity differences. Moreover, gender role attitudes may also intersect with factors such as generational position, educational background, or modernity orientation, and its experiential meaning does not fully correspond to specific cultural traditions or institutional arrangements.

Finally, this study does not adequately account for heterogeneity in attitudinal targets, such as the attitudinal differences between gay men and lesbian women, even though previous studies have demonstrated systematic differences in public acceptance of the two groups (87). In addition, this study does not explore how potential heterogeneity—such as urban–rural residence, generational cohort, religious affiliation, or local policy context—may moderate the key findings. The absence of subgroup heterogeneity analysis may obscure important differential effects across population segments. Future research may further differentiate between homosexual subgroups and incorporate reproductive values, and adopt longitudinal or mixed-method research designs, to better test the applicability of the family-oriented value framework proposed in this study across diverse social contexts.

## Data Availability

Publicly available datasets were analyzed in this study. This data can be found here: https://www.worldvaluessurvey.org/wvs.jsp.
